# Prevalence and Risk Factors Associated to Chronic Kidney Disease in HIV-Infected Patients on HAART and Undetectable Viral Load in Brazil

**DOI:** 10.1371/journal.pone.0026042

**Published:** 2011-10-12

**Authors:** Andréia M. Menezes, Jorge Torelly, Lúcia Real, Mônica Bay, Julia Poeta, Eduardo Sprinz

**Affiliations:** 1 Faculdade de Medicina, Universidade Federal do Rio Grande do Sul, Porto Alegre, Rio Grande do Sul, Brazil; 2 Hospital de Clínicas de Porto Alegre, Porto Alegre, Rio Grande do Sul, Brazil; University of Sao Paulo, Brazil

## Abstract

**Background:**

To determine the prevalence and associated factors with chronic kidney disease (CKD) in a cohort of HIV-positive individuals with undetectable viral load on HAART.

**Methods:**

From March, 2009 to September 2009, 213 individuals between 18-70 years, period on HAART ≥12 months, viral load < 50 copies/mm^3^, and CD4 ≥ 200 cells/mm^3^, were consecutively enrolled at the outpatient clinic of Hospital de Clínicas, Porto Alegre, Brazil. Exclusion criteria were obesity, malnourishment, amputee, paraplegic, previous history of renal disease, pregnancy and hepatic insufficiency. Renal function was determined by estimated glomerular filtration rate (eGFR) assessed by the modification of diet in renal disease. CKD was defined as an eGFR less or equal than 60 ml/min/1.73 m^2^, for a period of at least 3 months. Poisson regression was used to determine factors associated with CKD.

**Results:**

CKD was diagnosed in 8.4% of the population, and after adjustment, the risk factors were hypertension (RR = 3.88, 95%CI, 1.84 - 8.16), time on HAART (RR = 1.15, 95%CI,1.03–1.27) and tenofovir exposure (RR = 2.25, 95%CI, 1.04–4.95). Higher weight (RR = ,0.88 95%CI, 0.82–0.96) was associated to normal function.

**Conclusions:**

CKD was a common finding in this cohort of patients and was related to hypertension, time on HAART and tenofovir exposure. We suggest a more frequent monitoring of renal function, especially for those with risk factors to early identify renal impairment.

## Introduction

The advances of antiretroviral therapy (HAART) had turned HIV/AIDS into a chronic disease [1]–[5]. As a consequence of living longer, individuals might present complications not only related to the virus, but also related to the treatment itself and ageing process [1]–[4]. These complications might be related to heart, lung, hepatic or renal diseases, for instance, which could be associated to a higher mortality [2], [3].

In this scenario, kidney disease has been increasingly reported, with a prevalence of chronic kidney disease (CKD), varying from 4.7% to 7.6% according to several studies [5]–[8]. Risks that have already been associated include the “traditional” factors related to ageing, diabetes mellitus (DM) and hypertension [5], [6]. Besides that, other features such as the virus itself and some of the antiretrovirals (ARVs) currently used, such as indinavir (IDV) and tenofovir (TDF) have already been linked with nephrotoxicity in these individuals [5], [7], [8].

The first stage of renal impairment is silent and only detectable through laboratory analysis [9]. Although the most used biomarker for renal dysfunction diagnosis, the measurement of serum creatinine has limitations, such as the relatively low sensitivity, which underestimates the underlying renal status, and therefore, its values could be in the “normal range”, even after the loss of 50–60% of the glomerular filtration rate (GFR) [9], [10]. Although not clearly validated to HIV-infected individuals, equations adjusting for muscle mass, age, weight, ethnicity and sex, provide a more sensitive estimation of the true renal function, and have been used in general population (eGFR)[11], [12]. Currently, the most common formulas to estimate the GFR are Modification of Diet in Renal Disease formula (MDRD) [13], [14], Cockcroft-Galt (CG) [14], [15] and Chronic Kidney Disease Epidemiology Collaboration (CKD-epi) [16].

The objectives of our study were to describe the prevalence of CKD and to determine the risk factors associated to the development of renal disease in a cohort of HIV infected individuals on HAART, undetectable viral load, and not severely immunosupressed (CD4 count > 200 cells/mm3), which would better resemble the general population, in Brazil.

## Materials and Methods

### Population

Two hundred fifty five patients attended in the HIV/AIDS outpatient clinic at Hospital de Clínicas, Porto Alegre, Brazil, were consecutively enrolled from March, 2009 through September, 2009. To be included, patients needed to be on HAART and undetectable viral load (<50 copies/ml) for a period equal or greater than 12 months, CD4 counts higher than 200 cells/mm3 and age between 18 and 70 years. Patients were excluded if were obese or malnourishment [17], [18] due to the MDRD equation; amputee and paraplegic (with decreased muscle mass), previous history of kidney disease; present history of hepatitis B or C infection (confirmed by a positive polymerase chain reaction) or hepatic insufficiency; and, pregnant women.

## Methods

Data were collected at the moment of the appointment and included demographic variables (ethnicity, height, weight, BMI, sex and age); diagnosis of DM (according to “The American Diabetes Association”)[19], use of antidiabetic agents; hypertension (according to the Joint National Comittee 7 [20]); a complete history of HAART; use of drugs for prophylaxis against opportunistic infections; CD4 cell counts (cells/mm^3^, measured by flow cytometry); plasma HIV-RNA level (copies/ml, measured by b-DNA; HIV-1 RNA 3.0 assay, with limit of detection of 50 copies/ml); serum creatinine (mg/dl, measured by Jaffé; calibrated method by isotope dilution mass spectrometry - IDMS); date of HIV diagnosis and other comorbidities.

The biochemical data was measured in the same laboratory. The main outcome of interest in our study was to eGFR assessed by two measurements, at least, three months apart utilizing the MDRD in its simplified version (which considers serum creatinine concentration, age, sex and ethnicity) and CKD-epi equations. CKD was defined as an eGFR less or equal than 60 ml/min per 1.73 m^2^ or the presence of proteinuria independent of eGFR in both estimations.

Renal function was classified in five stages depending on the level of eGFR, as proposed by the Kidney Disease Outcomes Quality Initiative (KDOQI): normal, GFR equal or higher 90 ml/min per 1.73 m^2^; mild decrease, GFR between 60–89 ml/min per 1.73 m^2^ or presence of proteinuria with normal eGFR; moderate decrease, GFR between 30-59 ml/min per 1.73 m^2^; severe decrease, GFR between 15–29 ml/min per 1.73 m^2^; and, renal failure or dialysis, GFR less than 15 ml/min per 1.73 m^2^ [12].

The study was approved by the Research and Ethic Committee of Hospital de Clinicas and all patients signed the informed consent.

### Statistical analysis

Statistical analysis included descriptive (mean and standard deviation), univariate and multivariate analysis. Absolute and relative frequencies were utilized for continuous and categorical variables respectively.

To evaluate the association between CKD and categorical variables Chi-square test or Fisher exact were applied as required. Independent T test or one way ANOVA were used to compare means; in case of asymmetry Mann Whitney test or Kruskal-Wallis test were used. Variables significantly associated with renal impairment in univariate analysis (p<0.05) were included in the multivariate model. Those significant in the multivariate model (p<0.1) were included in the final model. Poisson Regression was used to determine the factors associated with CKD in an univariate analysis and in a multivariate model. Pearson coefficient and Bland-Altman graph were used to evaluate the correlation and the concordance between the two equations to eGFR (MDRD and CKD-epi). A p value of less than 0.05 was considered significant. Analysis was performed using the statistical package for the social sciences (SPSS) version 17.0 (SPSS Inc., Chicago, Illinois, USA).

### Sample size

The sample size calculation was based on a prevalence of at least 4% of CKD in that population with a confidence level of 95%. A sample size of at least 198 individuals was needed to be representative of the 2,050 individuals on HAART in our site to have a power of 80%

## Results

From the initial 255 included patients, there were 42 losses (16.5%): 28 due to incomplete laboratory data; 13 withdrew the informed consent; and, one additional patient died after signing the consent. The characteristics and clinical variables of the remained 213 patients are shown in Table 1. One hundred and ten patients were men (51.6%), 174 were euro-descendants (81.7%) and mean age was 45.6 years old, BMI was 24.75±4.05; mean time on HAART was 7.7±4.7 years, mean CD4+ count was 568±269 cells/mm^3^, and mean nadir CD4 count was 169±137 cells/mm^3^. The prevalence of hypertension and DM was 20.7% (n = 44) and 14.1% (n = 30), respectively. There was no statistical difference between ethnicity characteristics (data not shown).

**Figure 1:**
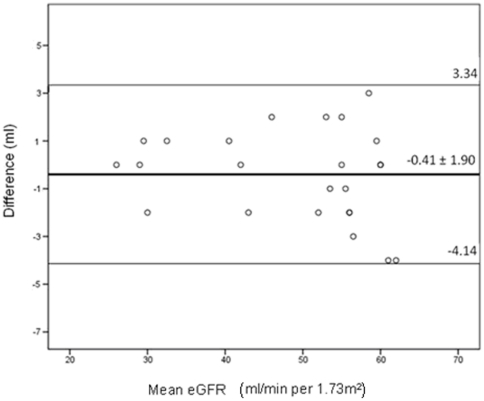
Bland-Altman graph comparing MDRD and CKD-epi in individuals with chronic kidney disease. There was a concordance in eGFR between the MDRD and CKD-epi equations in individuals with chronic kidney disease (the mean difference was 0.41±1.90 ml). eGFR  =  estimated glomerular filtration rate; MDRD  =  modified diet in renal disease; CKD-epi  =  chronic kidney disease epidemiology.

There was a significant correlation between the CKD-epi and MDRD equations (Pearson coefficient  =  0.9; p<0.001; Bland-Altman graph analysis p = 0.296; 95% IC -4.14–3.34, Figure 1). The mean eGFR was 98.04±30.35 ml/min/1.73 m2 for MDRD and 96.37±24.46 ml/min/1.73 m2 for CKD-epi (Table 2 shows the eGFR by MDRD equation of the cohort).

**Table 1:** Main demographic characteristics (data are presented as mean ± standard deviation or percentage).

	N = 213 (%)
Age (years)	45.6±11,5
Men	110 (51.6 %)
Ethnicity	
* Euro-descendants*	174 (81.7%)
Creatinine (mg/dl)	0.89±0.32
Urea (mg/dl)	35.44 ±13.34
eGFR^a^ (ml/mim)	98.04±30.35
CD4 (cells/mm^3^)	569.81±269
Hypertension	44 (20.7%)
Diabetes Mellitus	30 (14.1%)
Time on HAART^b^ (years)	7.8±4.8

a Estimated glomerular filtration rate,

b Antiretroviral therapy

**Table 2:** Prevalence of alteration in the renal function according to Kidney Disease Outcomes Quality Initiative – KDOQI [12], assessed by the modification of diet in renal disease.

Rena Function	eGFR^a^ (ml/min/1.73m^2^)	Our findings
		N = 213 (%)
Normal	> or equal 90	127 (59.6)
Mild Reduction	60–89	68 (31.9)
Moderate Reduction	30–59	15 (7)
Severe Reduction	15–29	3 (1.4)
Renal failure or dialysis	<15	0

a Estimated Glomerular filtration rate

The factors significantly associated with CKD in the univariate models (table 3) were: hypertension, DM, time after HIV diagnosis, older age, body weight, time on HAART, TDF and ritonavir (RTV) exposure (100 mg/day). After adjustment by the multivariate analysis (table 3), hypertension (RR = 3.88, 95% CI 1.84–8.16, p = <0.001), time on HAART (RR = 1.15, 95% CI 1.03–1.27, p =  0.011), and TDF exposure (RR = 2.25, 95% CI 1.04–4.95, p = 0.038) remained significantly associated to CKD. Higher weight (RR = 0.89, 95% CI 0.82–0.96, p = 0.005) was significantly associated to a normal kidney function.

**Table 3:** Risk factors associated to alteration in the renal function (eGFR < 60 ml/min per 1.73 m^2^ by MDRD); univariate and multivariate analysis.

	Univariate analysis			Multivariate analysis		
	RR	95% CI	P	RR	95% CI	p
Euro-descendants	5.15	0.78–37.03	0.103			
Men	1.06	0.50–2.26	0.864			
Use of atazanavir	2.03	0.87–4.70	0.098			
**Use of tenofovir**	**2.49**	**1.18–5.22**	**0.016**	**2.25**	**1.04–4.95**	**0,038**
Use of ritonavir (100mg)	3.88	1.66**–**9.03	0.02			
Use of ritonavir (200mg)	1.94	0.67–5.59	0.23			
**Hypertension**	**5.37**	**2.56–11.21**	**<0.001**	**3.88**	**1.84–8.16**	**<0.001**
Diabetes Mellitus	1.26	1.13**–**2.37	0.026			
Older age (years)	1.069	1.04–1.10	<0.001			
**Time on HAART (years)**	**1.14**	**1.06–1.23**	**<0.001**	**1.15**	**1.03–1.27**	**0.011**
**Body weight (Kg)**	**0.92**	**0.86–0.97**	**0.038**	**0.89**	**0.82–0.96**	**0.005**
Time of diagnosis of HIV	0.910	0.68**–**1.9	0.031	1.07	0.98**–**1.17	0.11
CD4 (cells/mm^3^)	1.0	0.99–1.01	0.97			

CKD defined as confirmed (persisting for ≤ 3 months) decrease in eGFR to 60 ml/min per 1.73m^2^ by MDRD or the presence of proteinuria independent of eGFR. CI, confidence interval; RR, risk ratio; eGFR, estimated glomerular filtration rate. All variables significant in univariate analyses (P<0.05) were included in multivariate model. All variables with P<0.1 in the multivariate analysis (data not shown) were included in the final model

## Discussion

Our study examined the prevalence of CKD and its associated factors among HIV infected individuals on HAART for at least one year and undetectable viral load (which is a quite clinically stable population). The prevalence of CKD was 8.4%, which is quite close, albeit higher, to the ones reported by other studies that vary from 4.7% to 7.6% [21]–[26]. Although somehow expected, this finding was worrisome for us, as it is estimated that about 5% of the general Brazilian population older than 60 years is expected to have CKD, whereas our cohort had a greater prevalence with CKD with a mean age almost 15 years younger (mean age was 45.6 years) [27],[28]. Likewise, as we selected HIV “healthy” patients (undetectable viral load and CD4 > 200 cells/3) and without previous renal disease we were anticipating a lower prevalence of CKD.

The factors associated were: hypertension, time on HAART and TDF use. As expected, hypertension was found to be a major risk factor associated with CKD (RR =  3.88, 95% CI 1.84–8.16)), which is in accordance to other studies [5], [6]–[8], [22], [24]. The risk was slightly higher than the other studies, which varied from 1.68% to 3.8% [5], [6], [8].

Another unique finding was that time on antiretroviral therapy was significantly associated with CKD. In our study, there was a 15% increased prevalence of CKD per year of additional exposure to ARVs (RR = 1.15, 95% CI 1.03–1.27). This could be explained by the fact that prolonged use of HAART could be associated with greater long term renal toxicity, meaning the more exposition leading to higher toxicity.

Adverse effects of the exposure to individuals ARVs were demonstrated by other studies which described the cumulative association of TDF, ATV, indinavir and RTV to the development of CKD [6], [8]. In our study, the only antiretroviral that was independently associated to CKD, was TDF (RR = 2.25, 95% CI 1.04–4.95). Our findings were somewhat higher than what have been found in other studies which varied from 1.5 to 2.18 higher chance of renal disease [5], [6], [8]. We did not have enough patients on indinavir (only 2) to find any association. Perhaps with a larger sample we could come across major findings regarding ritonavir use and, consequently, atazanavir, as it is the antiretroviral used along with 100 mg of ritonavir daily.

An important finding was related to the body weight (RR = 0.89, 95% CI 0.82–0.96, which was significantly associated as a protection factor. To our knowledge, this is the first study that found this association. Individuals with lower body weight were at greater risk of having CKD. Despite it is known that weight may play an important function in drug metabolism, once it can influence the bioavailability and pharmacokinetics of ARVs [29], there is still limited data about the effect of weight in HAART. Perhaps this should be a window of opportunity to individualize ARVs dosage and minimize toxicity.

The study has several limitations that should be considered when interpreting the results. Although all studies estimate the GFR in HIV individuals, this could be not accurate enough to allow a firm conclusion of kidney function. MDRD equation, generally more accurate and utilized [11], [13], might not be fully applicable to specific populations. Limited data suggests that MDRD may underestimate GFR in individuals with normal renal function [11], [13], [16] and therefore have led to an overestimation of renal impairment. We suggest that there is a need of an individualized equation to estimate the GFR in each population. Likely, due to constraints of MDRD, we excluded malnourished and obese patients, which could have better contributed to some of our findings. Secondly, our sample size might have limited our findings as it may have not been large enough to detect all CKD risk factors in this population, such as age and DM. Thirdly, this is a cross sectional study and therefore we can only draw association of events and not establish temporal sequence. Lastly, although we tried to minimize HIV infection and its co-morbidities, selecting individuals with CD4 counts greater than 200 cells/mm^3^ and at least one year with undetectable viral load, it is not possible to exclude any influence of HIV infection and related diseases or other nephrotoxic medications in the prevalence of renal impairment [5].

In summary, according to our findings, in HIV population which would most resemble the general population, hypertension, time on HAART, and exposure to tenofovir were associated with a higher prevalence of CKD. On the other hand, individuals with higher body weight appeared to be “protected” from chronic kidney disease. We suggest that an equation to eGFR should be routinely applied to better identify decrease in renal function, and should be performed at the moment of HIV diagnosis and later at regular intervals, depending on the associated risk factors. Further studies are necessary to confirm risks associated to renal disease in HIV-infected individuals.
